# Tracing the Geographical Origin of Durum Wheat by FT-NIR Spectroscopy

**DOI:** 10.3390/foods8100450

**Published:** 2019-10-02

**Authors:** Annalisa De Girolamo, Marina Cortese, Salvatore Cervellieri, Vincenzo Lippolis, Michelangelo Pascale, Antonio Francesco Logrieco, Michele Suman

**Affiliations:** 1 Institute of Sciences of Food Production (ISPA), CNR-National Research Council of Italy, Via G. Amendola 122/O, 70126 Bari, Italy; marinacortese88@gmail.com (M.C.); salvatore.cervellieri@ispa.cnr.it (S.C.); vincenzo.lippolis@ispa.cnr.it (V.L.); michelangelo.pascale@ispa.cnr.it (M.P.); antonio.logrieco@ispa.cnr.it (A.F.L.); 2 Research Development & Quality, Barilla G. & R. Fratelli S.p.A., Via Mantova 166, 43100 Parma, Italy; Michele.Suman@barilla.com

**Keywords:** FT-NIR spectroscopy, durum wheat, geographical origin, linear discriminant analysis

## Abstract

Fourier transform near infrared (FT-NIR) spectroscopy, in combination with principal component-linear discriminant analysis (PC-LDA), was used for tracing the geographical origin of durum wheat samples. The classification model PC-LDA was applied to discriminate durum wheat samples originating from Northern, Central, and Southern Italy (*n* = 181), and to differentiate Italian durum wheat samples from those cultivated in other countries across the world (*n* = 134). Developed models were validated on a separated set of wheat samples. Different pre-treatments of spectral data and different spectral regions were selected and compared in terms of overall discrimination (OD) rates obtained in validation. The LDA models were able to correctly discriminate durum Italian wheat samples according to their geographical origin (i.e., North, Central, and South) with OD rates of up of 96.7%. Better results were obtained when LDA models were applied to the discrimination of Italian durum wheat samples from those originating from other countries across the world, having OD rates of up to 100%. The excellent results obtained herein clearly indicate the potential of FT-NIR spectroscopy to be used for the discrimination of durum wheat samples according to their geographical origin.

## 1. Introduction

Durum wheat (*Triticum durum* Desf.) is a cereal crop that represents only 8% of the total wheat crop. Different from common wheat (*Triticum aestivum*), which is cropped in several areas of the world, durum wheat is mainly cropped in the Mediterranean basin—characterized by a relatively dry climate, with hot days and cool nights during the growing season—contributing to about 60% of the world production of this crop. The European Union (EU) is the largest producer and consumer of durum wheat in the world, with production largely concentrated in Italy, Greece, and Spain, together accounting for about 80% of the total EU production. Canada is the second largest producer of durum wheat in the world, and is a major durum wheat exporter. The United States, North Africa, Turkey, Syria, Australia, Mexico, Kazakhstan, and India are also significant producers of durum wheat [1]. 

The peculiar characteristics of durum wheat, such as its large kernel size, hardness, and bright yellow color, along with its protein content and gluten strength, make it suitable for manufacturing a wide range of end products, such as pasta, bread, couscous, and bulgur. Among them, pasta is the main product of durum wheat technology and is a staple food in several different countries. Italy is the country with both the largest production (over 3.4 million tons) and consumption (25.3 kg per capita/year) of pasta. However, the Italian durum wheat crop is not sufficient in covering national needs; therefore, the industry utilizes both domestic Italian wheat as well as wheat imported from abroad [2]. Indeed, it has been estimated that Italy in 2016 produced approximately 3.9 million tons of durum wheat, yet imported more than 2 million tons of durum wheat selected from different countries throughout the world (United Nations data retrieval system, UNdata, http://data.un.org). Such a consistent level of imports serves not only to make up for the shortfall in quantity, but also to regulate the variation in quality of domestic crops in order to guarantee the quality of the end product. With the introduction of the “Made in Italy” trademark, identifying food products of very high quality that are processed and manufactured solely on Italian soil using solely Italian ingredients [3], the demand of Italian millers and pasta producers for domestic production is gradually increasing.

The geographical origin, the genotype, the climate conditions of the harvesting year (such as precipitation, temperature, and sunshine time), and the chemical composition of the soil can have effects on the wheat composition and thus on the quality of the final products [4]. This is one of the reasons why the characterization of geographical origin of a raw ingredient, or of a foodstuff, is gaining increasing interest from consumers and producers, especially when it is used for certification of quality, authenticity, or typicality. The authenticity of foods has two different aspects, that is, authenticity with respect to production (such as geographical and botanical origin, organic vs. nonorganic, fresh vs. frozen, wild vs. farmed) and authenticity with respect to the description reported on the label (e.g., adulteration issues). The increased reliance on the international trade of food ingredients, together with the increased industrialization of food processing, allows food fraud to continue to increase in scope, scale, and threat. At the same time, the expanding of the global market makes the consumer more concerned about the origin of the products they consume. In order to achieve a high level of protection for consumers and to guarantee that consumers are appropriately informed with regards the food they consume, the European commission has laid down rules for indicating the country of origin or place of provenance of the primary ingredient of a food [5]. Recently, the European Commission has implemented the current regulation to better clarify the rules for indicating the country of origin of a primary ingredient that, depending on the geographical area, should be given as “EU”, “non-EU”, or “EU and non-EU” [6]. 

Considering the current legislation and to prevent adulteration practices and mislabeling regarding geographical origin, reliable analytical methods are required to address authenticity problems. Several methods have been proposed for determination of geographic origin of food products, including wheat. In a recent review covering the period from late 2008 until early 2015, the methods used mainly comprised isotopic analysis, (semi-)nondestructive analysis, compositional analysis, and elemental analysis [7]. (Semi-)nondestructive methods refers to methods for which minimal or no sample preparation is required, and the sample is not destroyed by extraction. These features are characteristics of a range of spectroscopic methods including infrared (IR) spectroscopy working in the near (NIR) and middle (MIR) spectral ranges and hyperspectral imaging. Furthermore, IR spectroscopy, in combination with multivariate analysis, can enhance the information generated by the analysis of samples, allowing for a detection of a pattern in a data set, and for the development of mathematical models to monitor authenticity and traceability [8,9]. IR spectroscopy has been successfully applied to several applications in the field of identification of geographical origin, such as virgin olive oil, cheese, wine, honey, tea, lentils, distillers dried grains with solubles, and other food products [7,9,10,11,12,13]. However, only a few applications of IR spectroscopy to differentiate wheat samples for geographical origin have been described in the literature. In a first study, Zhao et al. [14] demonstrated the potential of using NIR spectroscopy in combination with chemometrics to discriminate common wheat samples originating from different regions in China. Then, the same authors demonstrated the effect of grown origin, genotype, and harvest year on NIR spectral fingerprints of common wheat [15]. A successful use of NIR spectroscopy was also described by Gonzalez-Martin et al. [16] and Wadood et al. [12] to discriminate common wheat and common wheat flours originating from different regions of Chile and China, respectively. However, considering the low number of NIR applications in this field, more data are needed to demonstrate the effectiveness of NIR spectroscopy to be used as a valid method for tracing the geographical origin of wheat.

In the light of these results reported in the literature, the aim of the present paper was to report the use of Fourier transform-NIR (FT-NIR) spectroscopy to discriminate durum wheat samples originating from different areas of Italy and to discriminate Italian durum wheat samples from those originating from other countries. This is the first time that FT-NIR spectroscopy has been used for these purposes and that durum wheat has been investigated.

## 2. Materials and Methods 

### 2.1. Durum Wheat Samples and FT-NIR Spectroscopy Analysis

Fifty-nine durum wheat (*Triticum durum*) samples (from 50 to approximately 120 g each) of the 2017–2018 crop season were collected from local Italian producers located in 11 different Italian regions, namely, Abruzzo, Apulia, Campania, Emilia Romagna, Lazio, Lombardy, Marche, Molise, Tuscany, Umbria, and Veneto. Official sampling of wheat samples were carried out by a certification body for Italian origin certification [17]. Furthermore, an additional 29 durum wheat samples (from 50 to approximately 120 g each) were imported from eight different foreign countries, namely, Australia, Canada, France, Greece, Russia, Spain, Turkey, and the United State. These selected foreign countries are among the most relevant local producers of durum wheat, apart from Italy itself. No data about merceological characteristics were available for collected samples. Each sample was finely ground by the Retsch ZM 200 (Retsch, Haan, Germany) laboratory mill, obtaining grounded samples with particle size ≤500 μm.

FT-NIR spectra were recorded using the spectrometer Nicolet iS50 FT-IR (Thermo Fisher Scientific Inc., Madison, WI, USA) equipped with an interferometer and an integrating sphere, working in diffuse reflection and with an indium gallium arsenide (InGaAs) detector. Approximately 15–20 g of grounded wheat samples were placed on the rotary sample-cup spinner, and spectra were recorded by using 32 interferometer sub-scans in the range from 10,000 to 4000 cm^−1^, with a resolution of 8 cm^−1^. The time analysis was approximately of 30 seconds. Absorbance data were collected with OMNIC software v 9.0 (Thermo Fisher Scientific Inc., Milwaukee, WI, USA).

### 2.2. Chemometric Analysis 

The FT-NIR spectral data were imported as OMNIC SPA files into The Unscrambler X, v10.1 (CAMO Software AS, Oslo, Norway, 2011) software in order to perform the multivariate statistical analysis. The principal component analysis (PCA) was performed on both raw and pre-processed spectra to explore data and to recognize potential clustering (similarities and differences) of wheat samples. The presence of outliers was evaluated by using the graphical tools of the Unscrambler X software, that is, the Hotelling T² line plot using a critical limit of *p*-value < 5% and the influence plot, displaying samples with high leverage. The assignment of FT-NIR wheat signals was done through comparison with the literature [12,14,15,16,18,19,20]. Before development and validation of chemometric models, the FT-NIR spectral data were pre-treated using mean normalization, detrending, or standard normal variate (SNV) to reduce the spectral baseline shift, noise, and light scatter influence. Pre-treated data of FT-NIR spectra were then used to classify wheat samples based on their certified geographical origin using principal components-linear discriminant analysis (PC-LDA). 

#### Wheat Sample Classification

Two different classifications models for geographical origin discrimination were built. In the first one, Italian wheat samples were classified into three geographical areas depending on the area in which they were grown. In particular, samples from Emilia Romagna, Lombardy, and Veneto were classified as belonging to “Northern” Italy; samples from Lazio, Marche, Tuscany, and Umbria as belonging to “Central” Italy; and samples from Abruzzo, Campania, Molise, and Apulia were classified as belonging to “Southern” Italy. Furthermore, in order to increase the number of samples and to balance the number of samples through the three areas, each sample was split in sub-samples (from two to six, depending on the available amount of wheat) that were acquired independently, and an overall set of 181 wheat samples (i.e., *n* = 56 for Northern; *n* = 61 for Central; *n* = 64 for Southern) was obtained (Table 1). 

The total of 181 samples were randomly divided into two data sets, that is, the calibration set, containing 121 wheat samples (*n* = 37 for Northern; *n* = 41 for Central; *n* = 43 for Southern), and the validation set, containing 60 wheat samples (*n* = 19 for Northern; *n*= 20 for Central; *n* = 21 for Southern). Both calibration and validation sets contained a balanced number of samples among the three classes. 

The second geographical origin classification was aimed at discriminating wheat samples from “Italy” against wheat samples collected across the world (i.e., Spain, Turkey, Greece, Russia, France, Australia, the United States, and Canada) that were put together in a unique class named “other countries”. To have a number of samples from “other countries” (*n* = 29) comparable to those from “Italy” (*n* = 59), each of the samples from “other countries” was split into several sub-samples (from two to six depending on the available amount) that were acquired independently. A total set of 75 wheat samples from “other countries” was obtained (Table 2). 

Then, samples from “other countries” (*n* = 75) and samples from “Italy” (*n* = 59) were randomly divided into the calibration set, containing 104 wheat samples (*n* = 44 for “Italy”; *n* = 60 for “other countries”), and the validation set, containing 30 wheat samples (*n* = 15 for “Italy”; *n* = 15 for “other countries”). Both calibration and validation sets contained a balanced number of samples among the two classes. Then, spectral data were pre-treated and used to classify wheat samples on the basis of their geographical origin using the PC-LDA. The first 15 principal components (PCs), accounting for more than 99% of the total variance, were selected as input variables for the LDA. 

The performance of the classification models developed for the data of FT-NIR were evaluated from the results of the analyses of the validation datasets and expressed in terms of overall discrimination (OD) rate, correctly classified (CC), and misclassified (MSC) samples. The OD rate was calculated as percentage value by considering the sum of correctly classified wheat samples in all classes with respect to the total number of samples; CC samples (%) in each class were calculated by considering the number of correctly classified samples in the class with respect to the number of samples of the respective class; MSC samples (%) were calculated by considering the number of misclassified samples in the class with respect to the number of samples of the respective class. The tested pre-treatments (i.e., mean normalization, detrending, or SNV) were compared in terms of OD rates and CC values.

## 3. Results and Discussion

### 3.1. NIR Spectra of Wheat: Assignment of Spectral Bands 

**Figure 1:**
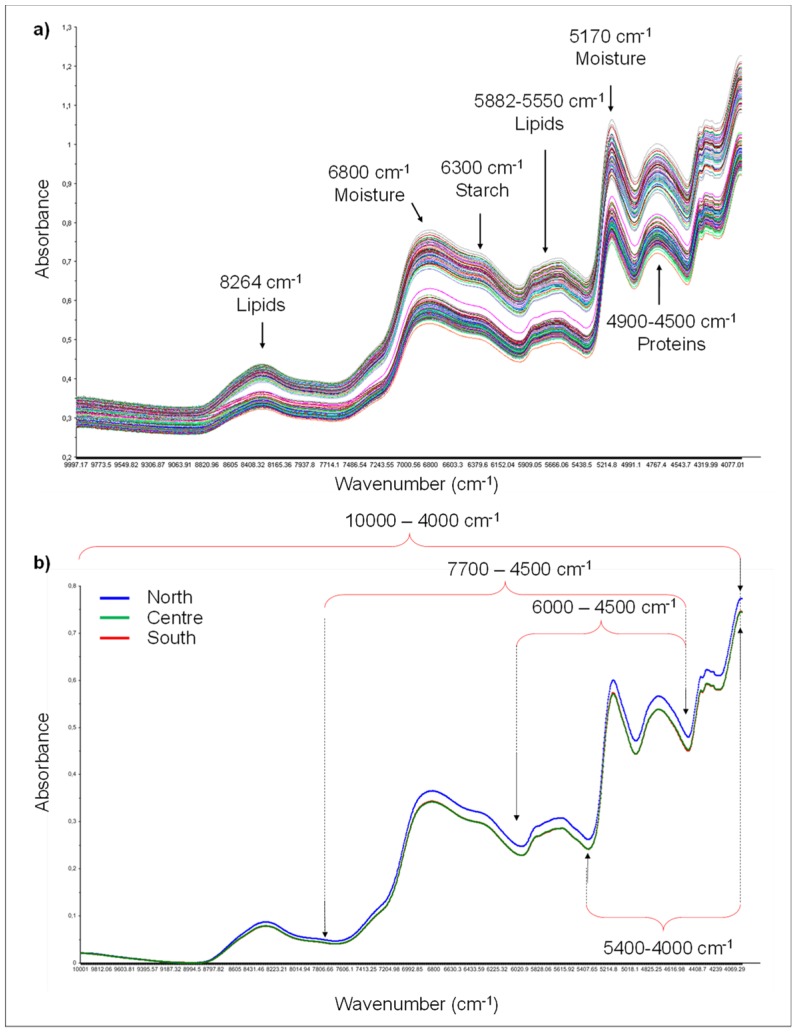
Raw Fourier transform near infrared (FT-NIR) spectra of the entire set of 181 Italian wheat samples with fundamental spectral bands (**a**), and average FT-NIR spectra of the Northern (blue trace), Central (green trace), and Southern (red trace) wheat classes. Central and Southern traces completely overlapped (**b**).

The overall raw Italian spectra and the average spectra of the classes Northern, Central, and Southern Italy are graphically shown in Figure 1a,b, respectively. All samples showed a similar trend in absorption area without large differences in the shape of spectra. The most noteworthy absorbance regions observed in the spectra had absorption bands around 8264 cm^−1^ (1200 nm), 6803 cm^−1^ (1470 nm), 6300 cm^−1^ (1587 nm), 5882 cm^−1^ (1762 nm), 5170 cm^−1^ (1934 nm), and 4900–4500 cm^−1^ (2040–2222 nm). The absorption band at 8264 cm^−1^ was related to the second overtone of C–H stretch related to lipids; the big absorbance peaks around 6803 cm^−1^ was related to the first overtone of O–H stretching and associated to moisture content; the small absorbance at 6300 cm^−1^ was related to the first overtone of O–H stretching and was associated with starches, whereas the small peak around 5882 cm^−1^ was related to the first overstone of C–H stretching and was associated with lipids; finally, the absorbance peaks between 4900–4500 cm^−1^ were related to a combination of C–H, N–H stretching, and O–H stretching, and were associated with proteins [18,19,20]. These noteworthy absorbance regions were in agreement with those reported elsewhere [12,14,15,16].

Because of this overlapping of peaks and broad spectral bands, the average spectra of wheat samples belonging to each class, namely, Northern, Central, and Southern Italy, were calculated and compared to try to visually differentiate samples on the basis of their geographical origin. As can be seen in Figure 1b, the mean spectra of the classes Central and Southern Italy were completely overlapped, while a signal amplification was observed in the average spectra of samples from Northern Italy, mainly in the region from 7000 to 4500 cm^−1^.

### 3.2. Developmnt and Validation of PC-LDA Models for Geographical Origin Discrimination of Durum Wheat

#### 3.2.1. PC-LDA Models for Classification of Durum Wheat Samples Collected from Northern, Central, and Southern Italy

**Figure 2:**
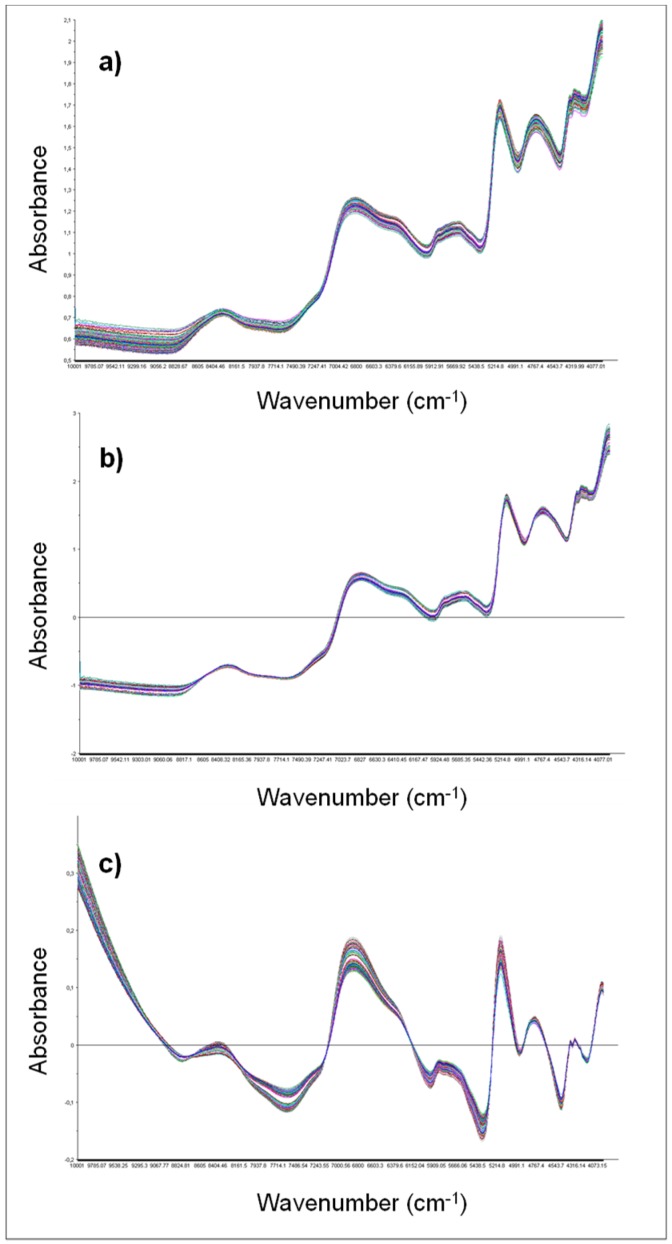
FT-NIR spectra of the entire set of Italian wheat samples pre-treated with mean normalization (**a**), standard normal variate (**b**), and detrending (**c**).

The spectral data were pre-treated using mean normalization, detrending, or SNV before the development and validation of classification models to reduce the spectral baseline shift, noise, and light scatter influence. A clear improvement of absorbance features of raw wheat spectra was observed after each pre-treatment (Figure 1a and Figure 2); for example, mean normalization reduced bias from the spectra and spectral distorsions because of the scattering between spectra, detrending eliminated variations in baseline shift, and curvilinearity, whereas SNV reduced the multiplicative interferences of scatter and particle size of raw spectra [19]. 

Then, because of the complexity of FT-NIR spectra and the little differences between wheat samples, it was essential to apply multivariate data analysis on pretreated spectral data to differentiate wheat samples on the basis of their geographical origin. Furthermore, other than the entire spectral range (i.e., from 10000 to 4000 cm^−1^), reduced spectral regions were singularly considered and compared to evaluate which of them favored the geographical origin discrimination of durum wheat. The most promising results were those obtained considering regions from 7700 to 4500 cm^−1^, from 6000 to 4500 cm^−1^, and from 5400 to 4000 cm^−1^) (Figure 1b). 

In a first step, principal component analysis (PCA) with full cross validation was applied to the whole set of 181 samples using the pre-treated FT-NIR spectra to extract information on the major trends in the whole set and to figure out a preliminary discrimination of wheat samples as a function of their geographic origin. In each case, the corresponding PCA score plots did not reveal any spatial pattern in the sample score distribution and only led to a slight discrimination of wheat samples grown in different parts of Italy when restricted spectral regions were used in place of the entire spectrum. 

In a second step, the classification tool principal components-linear discriminant analysis (PC-LDA) was applied to the pre-treated FT-NIR spectra to classify durum wheat samples on the basis of their geographical origin. The first 15 PCs of the PCA, accounting for more than 99% of the total variance, were used as input variables for the LDA. All the 181 Italian wheat samples were used to develop and validate PC-LDA models; in particular, a total of 121 samples was used for the calibration of the model and 60 samples for its validation. To select the best pre-treatment and spectral region able to discriminate wheat samples according to their geographical origin, validation results were compared in terms of overall discrimination (OD) rates. PC-LDA models always yielded good OD rates, ranging from 95.0% to 100% in calibration and from 90.0% to 96.7% in validation, depending on the pre-treatment applied to the considered range. The best validation results were those obtained by applying both the mean normalization baseline (OD between 93.3%–96.7%) or SNV (OD between 91.7%–96.7%) pre-treatments in the spectral range from 6000 to 4500 cm^−1^ (1666–2222 nm). These results were different from those reported by Zhao et al. [15] that selected, besides the wavelength ranges between 975–990 nm (that were not included in our equipment), the wavelength of 1200 nm and the range between 1355–1380 nm—as they contain enough information to develop robust discriminant models. 

**Figure 3:**
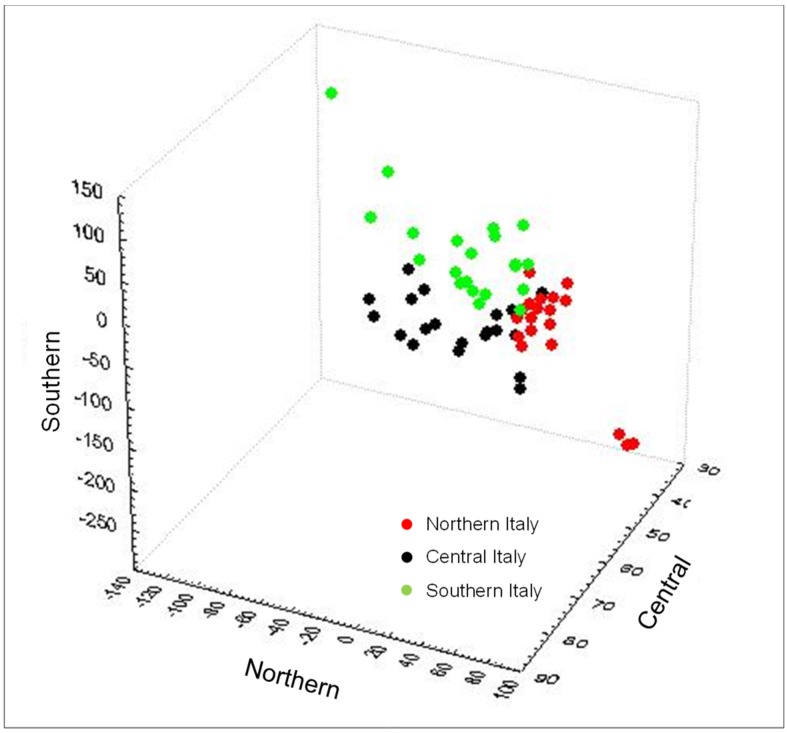
Principal components-linear discriminant analysis (PC-LDA) score plot for wheat samples (validation results) grown in different geographical areas of Italy (North, Central, and South) analyzed by FT-NIR in the spectral region between 6000–4500 cm^−1^ and using the mean normalization pre-treatment of spectral data. Samples from different geographical origin are presented by different symbols.

Detailed discrimination results of the PC-LDA models obtained in validation for wheat samples grown in different geographical areas of Italy (Northern, Central, and Southern) and analyzed in different FT-NIR spectral regions using the mean normalization pre-treatment are reported in Table 3, while the PC-LDA score plot is shown in Figure 3. It is noteworthy to mention that the class of wheat samples grown in the Southern Italy gave almost always a 100% correct classification in both calibration and validation, whereas some misclassifications occurred for wheat samples grown in Central and Northern Italy, potentially related to the climatic conditions that are humid and cold in Northern Italy and are generally warmer and dryer in Southern Italy, leading to the differentiation in chemical composition of the wheat. 

Results obtained herein were in agreement with those reported by other authors that applied the NIR spectroscopy in combination with chemometric analysis to the discrimination of common wheat grown from different regions of China [12,14,15] and Chile [16]. Other successful applications of NIR spectroscopy to the geographical discrimination of cereals and derived products include arabica coffee from Brazil [21,22] and corn distillers dried grains from various countries [13]. The majority of these papers combine NIR spectroscopy with the partial least squares discriminant analysis (PLS-DA) and, to lesser extent, with LDA chemometric model for the geographical discrimination of wheat. For example, Zhao et al. [14] applied both PLS-DA and LDA models for determination of geographic origin of common wheat kernels and wheat flour samples. Overall discrimination rates ranged from 85% to 95.5% for PLS-DA models and from 72.5% to 85.0% for LDA models. Considering the better results obtained with PLS-DA model, the same group of Zhao and co-workers [15] applied this chemometric model in a further study to the discrimination of common wheat kernels from China for their grown origin, genotype, and harvest year. Similarly, Gonzalez-Martin et al. [16] proposed PLS-DA models for discriminating common wheat kernels and durum wheat flours originating from different regions of Chile. Marquetti et al. [22] proposed a PLS-DA model that achieved in validation an OD rate of 94.4% for discriminating arabica coffee samples geographically and genotipically. On the other hand, in our study, we demonstrate the power and the effectiveness of the PC-LDA model to successfully discriminate wheat samples on the basis of their geographical origin. Indeed the PC-LDA score plot showed a good separation between the three Italian geographic regions, even though classes “Northern” and “Southern” were more scattered compared with “Central” Italy (Figure 3). Results obtained herein were in agreement with those described by Wadood et al. [12] who applied the LDA chemometric model to discriminate wheat kernels and wheat flour for their geographical origin and production year. In this case, OD rates ranged from 61.1% to 100%, with wheat flour providing the best results. 

#### 3.2.2. PC-LDA Models for Classification of Durum Wheat Samples Grown in Italy and in Several Other Countries across the World

As previously observed for FT-NIR spectra of Italian durum wheat samples, it was also observed that in the wheat samples originating from different countries across the world (i.e., Spain, Turkey, Greece, Russia, France, Australia, the United States, and Canada) the same noteworthy absorbance regions existed around 8264 cm^−1^, 6803 cm^−1^, 6300 cm^−1^, 5882 cm^−1^, 5170 cm^−1^, and 4900–4500 cm^−1^, thus confirming previously reported results [12,14,15,16]. The spectral data of wheat samples grown in several other countries across the world were then pre-treated using mean normalization, detrending, or SNV, as previously done with wheat samples grown in Italy. As expected, a clear improvement of absorbance features of raw wheat spectra was observed after these pre-treatments. Then, the multivariate statistical analysis was applied to FT-NIR spectra to discriminate wheat samples grown in Italy from those grown in other different eight countries across the world, which are often mixed with locally produced wheat. 

**Figure 4:**
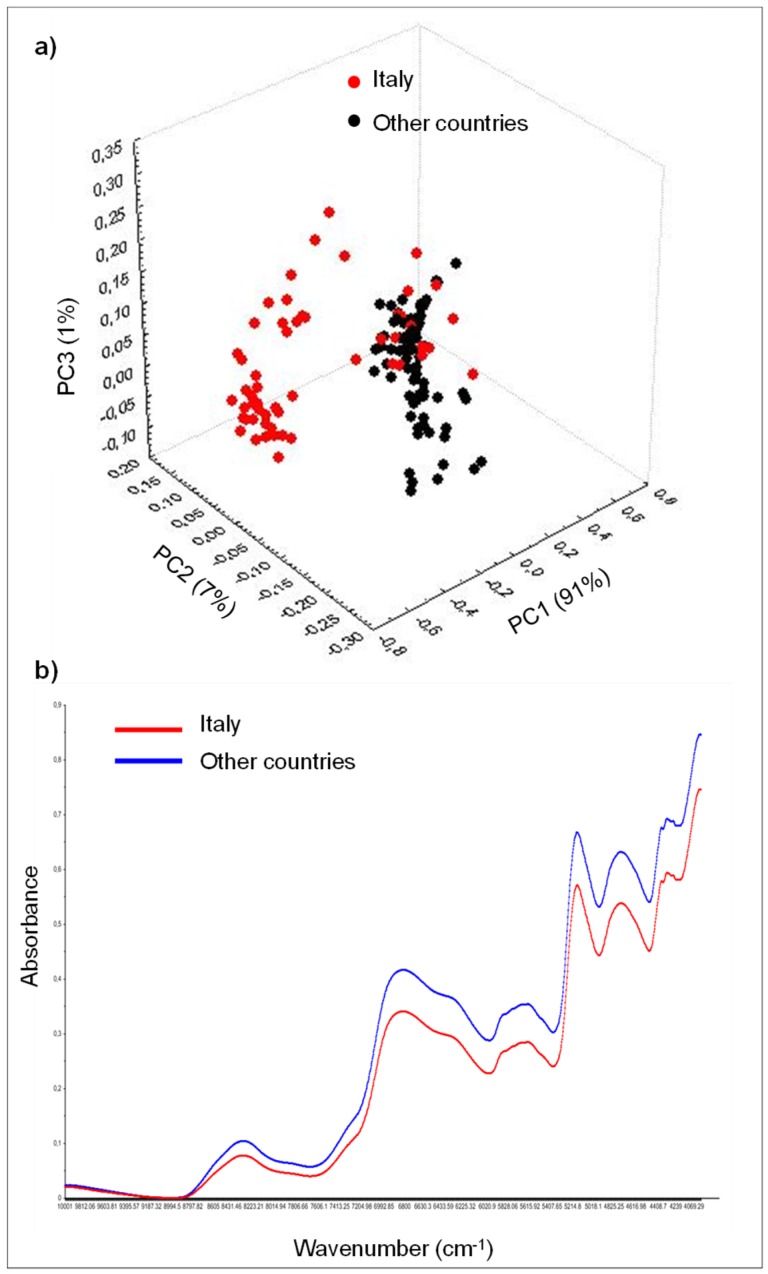
Average Principal component analysis (PCA) score plot for the entire set of wheat samples grown in “Italy” and in “other countries” across the world, analyzed by FT-NIR in the spectral region between 10000–4000 cm^−1^ and using the detrending pre-treatment of spectral data. Samples from different geographical origin are presented by different symbols (**a**). FT-NIR spectra of wheat classes “Italy” and “other countries” (**b**).

The PCA with full cross validation was applied to the pre-treated FT-NIR spectra of the whole set of 134 wheat samples (i.e., 59 from “Italy” and 75 from “other countries”). The entire spectral region (i.e., from 10,000 to 4000 cm^−1^), as well as the reduced spectral regions (i.e., from 7700 to 4500 cm^−1^, from 6000 to 4500 cm^−1^, and from 5400 to 4000 cm^−1^) were investigated and compared as previously done for Italian wheat samples. In each case, the corresponding PCA score plot revealed two major clusters of samples corresponding to wheat samples grown in Italy and wheat samples grown in “other countries”, respectively (Figure 4a). This separation between the origin was also evident by comparing the average spectra of the two classes (“Italy” and “other countries”) in the spectral region between 7000 and 4500 cm^−1^, related to the content of starch, lipid, and proteins that, in the case of wheat grown abroad of Italy, were more rich (Figure 4b). 

Then, the PC-LDA chemometric tool was applied to the pre-treated FT-NIR spectra using the different spectral regions. The first 15 PCs of the PCA, accounting for more than 99% of the total variance, were used as input variables for the LDA. All the 134 wheat samples were used to develop and validate PC-LDA models; in particular, a total of 104 samples was used for the calibration of the models and 30 samples for their validation. PC-LDA models always yielded good OD rates that ranged from 93.3% to 100% in calibration and from 86.7% to 100% in validation, depending on the pre-treatment applied and the FT-NIR spectral region considered. The best results were those obtained by applying the detrending pre-treatment in the spectral region from 6000 to 4500 cm^−1^ and from 7700 to 4500 cm^−1^, as previously observed for discrimination of Italian samples into three geographical areas, together with the spectral region from 5500 to 4000 cm^−1^ (Table 4). 

**Figure 5:**
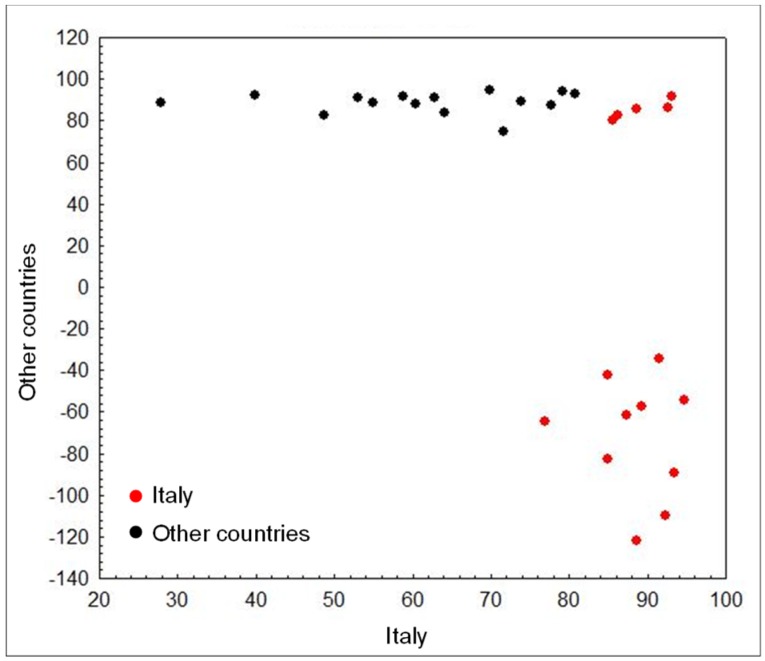
PC-LDA score plot for wheat samples (validation results) grown in “Italy” and in “other countries” across the world, analyzed by FT-NIR in the spectral region between 6000–4500 cm^−1^ and using the detrending pre-treatment of spectral data. Samples from different geographical origin are presented by different symbols.

The PC-LDA score plot showed a clear separation between the two classes. Furthermore, in the cluster of Italian samples, it was possible to see a smaller group of five wheat samples from Central–Southern Italy that, even if correctly classified, were close to wheat samples from France and Canada (Figure 5). 

The excellent results obtained herein clearly indicate the potential of FT-NIR spectroscopy to be used for the discrimination of samples from “Italy” against samples from “other countries” across the world. Other authors have described the application of infrared spectroscopy and near infrared microscopy to the discrimination of corn distillers dried grains originating from different countries [11,13,23]. To the best of our knowledge, this is the first time that a similar study has been conducted on durum wheat samples using FT-NIR spectroscopy. Other techniques, including isotope ratio mass spectrometry, high-resolution inductively coupled plasma mass spectrometry, and gas chromatography have been proposed for the geographical discrimination of durum wheat samples [24,25,26,27,28,29]. Although all these techniques are effective and showed their potential to be used for this purpose, all of them are destructive, time-consuming, and require expensive instrumentations and skilled personnel to perform the analysis, as compared with FT-NIR spectroscopy, which is rapid, easy-to-use, cost-effective, and does not require skilled personnel for the analysis.

## 4. Conclusions

In the last decade, food traceability has gained an increasing interest from both consumers and producers because the claim of the geographical origin of foodstuffs may be used as one of the criterion for certification of quality. 

In this study, two LDA models based on the use of FT-NIR spectroscopy were developed and validated to discriminate durum wheat samples on the basis of their geographical origin. In particular, the first LDA model was able to discriminate wheat samples originating from different Italian areas, namely Northern, Central, and Southern Italy. The evaluation of external validation results demonstrated the robustness and reliability of the model, having an overall discrimination rate of up to 97%. The second LDA model was able to discriminate Italian wheat samples from the samples originating from eight different countries across the world and grouped in a unique class of samples, having overall discrimination rates of up to 100%. However, these results should be further validated through the use of samples originating from different growing seasons. 

The existence of clearly distinct groups for wheat samples originating from the different regions of the same country, as well as from different countries, supports the use of FT-NIR spectroscopy for the characterization of Italian wheat samples according to their geographical origin. Furthermore, considering that the traceability of geographical origin of Italian wheat is very important for the “Made in Italy” brand and for the Italian food industry, the potential of using FT-NIR spectroscopy as a reliable, rapid, and easy-to-use method for the fingerprinting of Italian durum wheat is evident. 

## Figures and Tables

**Table 1:** Classification of durum wheat samples originating from different regions of Italy.

Classification	Region	Number of Samples
Northern	Emilia Romagna	18
	Lombardy	20
	Veneto	18
	Total	56
Central	Lazio	15
	Marche	16
	Tuscany	13
	Umbria	17
	Total	61
Southern	Abruzzo	12
	Campania	15
	Molise	17
	Apulia	20
	Total	64
	Overall total	181

**Table 2:** Classification of durum wheat samples collected from across the world.

Classification	Countries	Number of Samples
Other countries	Spain	3
	Turkey	11
	Greece	11
	Russia	8
	France	13
	Australia	9
	United States	8
	Canada	12
	Total	75

**Table 3:** Classification table (validation results) of the principal component-linear discriminant analysis for wheat samples grown in different geographical areas of Italy and analyses by FT-NIR spectroscopy. Spectral data were pre-treated using mean normalization.

Spectral Region	Assigned Class ^a^	Predicted Class ^a^		
		Northern	Central	Southern
10,000–4000 cm^−1^	Northern	17	0	0
	Central	1	18	0
	Southern	1	2	21
	CC samples (%) ^b^	89.5	90.0	100.0
	OD rate (%) ^c^	93.3		
7700–4500 cm^−1^	Northern	18	1	0
	Central	1	19	0
	Southern	0	0	21
	CC samples (%) ^b^	94.7	95.0	100
	OD rate (%) ^c^	96.7		
6000–4500 cm^−1^	Northern	18	1	0
	Central	1	19	0
	Southern	0	0	21
	CC samples (%) ^b^	94.7	95.0	100
	OD rate (%) ^c^	96.7		
5500–4000 cm^−1^	Northern	17	1	0
	Central	2	19	1
	Southern	0	0	20
	CC samples (%) ^b^	89.5	95.0	95.2
	OD rate (%) ^c^	93.3		

^a^ Northern Italy: samples from Emilia Romagna, Lombardy, Veneto; Central Italy: samples from Lazio, Marche, Toscana, Umbria; Southern Italy: samples from Abruzzo, Campania, Molise, Puglia; ^b^ CC, correctly classified; ^c^ OD, overall discrimination.

**Table 4:** Classification table (validation results) of the principal component-linear discriminant analysis for wheat samples grown in “Italy” and in several “other countries” across the world, and analyzed by FT-NIR spectroscopy. Spectral data were pre-treated using detrending.

Spectral Region	Assigned Class ^a^	Predicted Class ^a^	
		Italy	Other Countries
10,000–4000 cm^−1^	Italy	13	1
	Other countries	2	14
	CC samples (%) ^b^	86.7	93.3
	OD rate (%) ^c^	90.0	
7700–4500 cm^−1^	Italy	15	1
	Other countries	0	14
	CC samples (%) ^b^	100	93.3
	OD rate (%) ^c^	96.7	
6000–4500 cm^−1^	Italy	15	0
	Other countries	0	15
	CC samples (%) ^b^	100	100
	OD rate (%) ^c^	100	
5500–4000 cm^−1^	Italy	15	1
	Other countries	0	14
	CC samples (%) ^b^	100	93.3
	OD rate (%) ^c^	96.7	

^a^ Italy: samples from different areas, i.e., Emilia Romagna, Lombardy, Veneto, Lazio, Marche, Toscana, Umbria, Abruzzo, Campania, Molise, and Puglia; other countries: samples from Spain, Turkey, Greece, Russia, France, Australia, the United States, and Canada; ^b^ CC, correctly classified; ^c^ OD, overall discrimination.

## References

[1] Grant C.A., Di Fonzo N., Pisante M., Sissons M., Abecassis J., Marchylo B., Carcea M. (2012). Durum Wheat: Chemistry and Technology.

[2] Ranieri R. (2015). Geography of the durum wheat crop. Pastaria Int..

[3] Italian Law (2009). LEGGE 20 Novembre 2009, n. 166. Conversione in Legge, Con Modificazioni, del Decreto-Legge 25 Settembre 2009, n. 135, Recante Disposizioni Urgenti per L’attuazione di Obblighi Comunitari e per L’esecuzione di Sentenze Della Corte di Giustizia Delle Comunità Europee. (09G0180).

[4] Sissons M. (2008). Role of durum wheat composition on the quality of pasta and bread. Food Glob. Sci. Book.

[5] European Commission (2011). Regulation (EU) No 1169/2011 of the European Parliament and of the Council of 25 October 2011 on the provision of food information to consumers, amending Regulations (EC) No 1924/2006 and (EC) No 1925/2006 of the European Parliament and of the Council, and repealing Commission Directive 87/250/EEC, Council Directive 90/496/EEC, Commission Directive 1999/10/EC, Directive 2000/13/EC of the European Parliament and of the Council, Commission Directives 2002/67/EC and 2008/5/EC and Commission Regulation (EC) No 608/2004. Off. J..

[6] European Commission (2018). Commission Implementing Regulation (EU) 2018/775 of 28 May 2018 laying down rules for the application of Article 26(3) of Regulation (EU) No 1169/2011 of the European Parliament and of the Council on the provision of food information to consumers, as regards the rules for indicating the country of origin or place of provenance of the primary ingredient of a food. Off. J..

[7] Pustjens A.M., Muilwijk M., Weesepoel Y., van Ruth S.M. (2016). Advances in Food Authenticity Testing.

[8] Cozzolino D. (2014). An overview of the use of infrared spectroscopy and chemometrics in authenticity and traceability of cereals. Food Res. Int..

[9] Mendez J., Mendoza L., Cruz-Tirado J.P., Quevedo R. (2019). Trends in application of NIR and hyperspectral imaging for food authentication. Sci. Agropecu..

[10] Innamorato V., Longobardi F., Lippolis V., Cortese M., Logrieco A.F., Catucci L., Agostiano A., De Girolamo A. (2019). Tracing the geographical origin of lentils (*Lens culinaris* Medik.) by infrared spectroscopy and chemometrics. Int. J. Food Sci. Technol..

[11] Tena N., Boix A., von Holst C. (2015). Identification of botanical and geographical origin of distillers dried grains with solubles by near infrared microscopy. Food Control.

[12] Wadood S.A., Guao B., Zhang X., Wei Y. (2019). Geographical origin discrimination of wheat kernel and white flour using near-infrared reflectance spectroscopy fingerprinting coupled with chemometrics. Int. J. Food Sci. Technol..

[13] Zhou X., Yang Z., Haughey S.A., Galvin-King P., Han L. (2015). Classification the geographical origin of corn distillers dried grains with solubles by near infrared reflectance spectroscopy combined with chemometrics: A feasibility study. Food Chem..

[14] Zhao H., Guo B., Wei Y., Zhang B. (2013). Near infrared reflectance spectroscopy for determination of the geographical origin of wheat. Food Chem..

[15] Zhao H., Guo B., Wei Y., Zhang B. (2014). Effects of grown origin, genotype, harvest year, and their interactions of wheat kernels on near infrared spectral fingerprints for geographical traceability. Food Chem..

[16] Gonzalez-Martin M.I., Moncada G.W., Gonzalez-Perez C., San Martin N.Z., Lopez-Gonzalez F., Ortega I.L., Hernandez-Hierro J.M. (2014). Chilean flour and wheat grain: Tracing their origin using near infrared spectroscopy and chemometrics. Food Chem..

[17] European Commission (2014). Commission Regulation (EU) No 519/2014 of 16 May 2014 amending Regulation (EC) No 401/2006 as regards methods of sampling of large lots, spices and food supplements, performance criteria for T-2, HT-2 toxin and citrinin and screening methods of analysis. Off. J..

[18] Manley M., Baeten V., Sun D.-W. (2018). Modern Techniques for Food Authentication.

[19] Stuart B. (2004). Infrared Spectroscopy: Fundamentals and Applications.

[20] Manley M. (2014). Near-infrared spectroscopy and hyperspectral imaging: Non-destructive analysis of biological materials. Chem. Soc. Rev..

[21] Bona E., Marquetti I., Link J.V., Makimori G.Y.F., da Costa Arca V., Lemes A.L.G., Ferreira J.M.G., dos Santos Scholz M.B., Valderrama P., Poppi R.J. (2017). Support vector machines in tandem with infrared spectroscopy for geographical classification of green Arabica coffee. LWT Food Sci. Technol..

[22] Marquetti I., Link J.V., Lemes A.L.G., dos Santos Scholz M.B., Valderrama P., Bona E. (2016). Partial least square with discriminant analysis and near infrared spectroscopy for evaluation of geographic and genotypic origin of Arabica coffee. Comput. Electron. Agric..

[23] Nietner T., Pfister M., Glomb M.A., Fauhl-Hassek C. (2013). Authentication of the botanical and geographical origin of distillers dried grains and solubles (DDGS) by FT-IR Spectroscopy. J. Agric. Food. Chem..

[24] Armanino C., De Acutis R., Festa M.R. (2002). Wheat lipids to discriminate species, varieties, geographical origins and crop years. Anal. Chim. Acta.

[25] Brescia M.A., Di Martino G., Guillou C., Reniero F., Sacco A., Serra F. (2002). Differentiation of the geographical origin of durum wheat semolina samples on the basis of isotopic composition. Rapid Commun. Mass Spectr..

[26] Dong H., Xiao K., Xian Y., Wu Y. (2018). Authenticity determination of honeys with non-extractable proteins by means of elemental analyzer (EA) and liquid chromatography (LC) coupled to isotope ratio mass spectroscopy (IRMS). Food Chem..

[27] Luo D., Dong H., Luo H., Xian Y., Wan J., Guo X., Wu Y. (2015). The application of stable isotope ratio analysis to determine the geographical origin of wheat. Food Chem..

[28] Wadood S.A., Boli G., Yimin W. (2018). Geographical traceability of wheat and its products using multielement light stable Isotopes coupled with chemometrics. J. Mass Spectr..

[29] Zhao H., Guo B., Wei Y., Zhang B., Sun S., Zhang L., Yan J. (2011). Determining the geographical origin of wheat using multielement analysis and multivariate statistics. J. Agric. Food Chem..

